# Effectiveness and safety of primary prophylaxis with G-CSF after induction therapy for acute myeloid leukemia: a systematic review and meta-analysis of the clinical practice guidelines for the use of G-CSF 2022 from the Japan society of clinical oncology

**DOI:** 10.1007/s10147-023-02465-0

**Published:** 2024-03-18

**Authors:** Tomoya Maeda, Yuho Najima, Yutaro Kamiyama, Shinji Nakao, Yukinori Ozaki, Hiroshi Nishio, Kenji Tsuchihashi, Eiki Ichihara, Yuji Miumra, Makoto Endo, Dai Maruyama, Tatsuhiro Yoshinami, Nobuyuki Susumu, Munetaka Takekuma, Takashi Motohashi, Mamoru Ito, Eishi Baba, Nobuaki Ochi, Toshio Kubo, Keita Uchino, Takahiro Kimura, Shinobu Tamura, Hitomi Nishimoto, Yasuhisa Kato, Atsushi Sato, Toshimi Takano, Shingo Yano

**Affiliations:** 1 Department of Hemato-Oncology, Saitama Medical University International Medical Center, 1397-1 Yamane, Hidaka, Saitama 350-1298 Japan; 2 Hematology Division, Tokyo Metropolitan Cancer and Infectious Diseases Center, Komagome Hospital, 3-18-22 Honkomagome, Bunkyo-Ku, Tokyo, 113-8677 Japan; 3 Division of Clinical Oncology and Hematology, Department of Internal Medicine, The Jikei University School of Medicine, 3-25-8 Nishi-Shinbashi, Minato-Ku, Tokyo, 105-8461 Japan; 4 Department of Hematology, Faculty of Medicine, Institute of Medical Pharmaceutical and Health Sciences, Kanazawa University, 13-1 Takaramachi, Kanazawa, Ishikawa, 920-8640 Japan; 5 Department of Breast Medical Oncology, The Cancer Institute Hospital of Japanese Foundation for Cancer Research, 3-8-31 Ariake, Koto-Ku, Tokyo, 135-8850 Japan; 6 Department of Obstetrics and Gynecology, Keio University School of Medicine, 35 Shinanomachi, Shinjuku-Ku, Tokyo, 160-8582 Japan; 7 Department of Hematology, Oncology and Cardiovascular Medicine, Kyushu University Hospital, 3-1-1 Maidashi, Higashi-Ku, Fukuoka, 812-8582 Japan; 8 Center for Clinical Oncology, Okayama University Hospital, 2-5-1 Shikata-Cho, Kita-Ku, Okayama, 700-8558 Japan; 9 Department of Medical Oncology, Toranomon Hospital, 2-2-2 Toranomon, Minato-Ku, Tokyo, 105-8470 Japan; 10 Department of Orthopedic Surgery, Kyushu University Hospital, 3-1-1 Maidashi, Higashi-Ku, Fukuoka, 812-8582 Japan; 11 Department of Hematology Oncology, The Cancer Institute Hospital of Japanese Foundation for Cancer Research, 3-8-31 Ariake, Koto-Ku, Tokyo, 135-8850 Japan; 12 Department of Breast and Endocrine Surgery, Graduate School of Medicine, Osaka University, Suita, Osaka 565-0871 Japan; 13 Department of Obstetrics and Gynecology, International University of Health and Welfare Narita Hospital, 4-3 Kozunomori, Narita, Chiba 286-8686 Japan; 14 Department of Gynecology, Shizuoka Cancer Center Hospital, Sunto-Gun, Shizuoka, 411-8777 Japan; 15 Department of Obstetrics and Gynecology, Tokyo Women’s Medical University Hospital, 8-1 Kawada-Cho, Shinjyuku-Ku, Tokyo, 162-8666 Japan; 16 Department of Oncology and Social Medicine, Graduate School of Medical Sciences, Kyushu University, 3-1-1 Maidashi, Higashi-Ku, Fukuoka, 812-8582 Japan; 17 Department of General Internal Medicine 4, Kawasaki Medical School, 2-6-1 Nakasange, Kita-Ku, Okayama, 700-8505 Japan; 18 Department of Allergy and Respiratory Medicine, Okayama University Hospital, 2-5-1 Shikata-Cho, Kita-Ku, Okayama, 700-8558 Japan; 19 Department of Medical Oncology, NTT Medical Center Tokyo, 5-9-22 Higashi-Gotanda, Shinagawa-Ku, Tokyo, 141-8625 Japan; 20 Department of Urology, The Jikei University School of Medicine, 3-25-8 Nishi-Shinbashi, Minato-Ku, Tokyo, 105-8461 Japan; 21 Department of Hematology/Oncology, Wakayama Medical University, 811-1 Kimiidera, Wakayama, 641-8509 Japan; 22 Department of Nursing, Okayama University Hospital, 2-5-1 Shikata-Cho, Kita-Ku, Okayama, 700-8558 Japan; 23 Department of Drug Information, Faculty of Pharmaceutical Sciences, Shonan University of Medical Sciences, 16–48 Kamishinano, Totsuka-Ku, Yokohama, Kanagawa 224-0806 Japan; 24 Department of Medical Oncology, Hirosaki University Graduate School of Medicine, 5 Zaifu-Cho, Hirosaki, Aomori, 036-8562 Japan

**Keywords:** G-CSF, AML, Adults, Neutropenia, Induction therapy, Meta-analysis

## Abstract

Although granulocyte colony-stimulating factor (G-CSF) reduces the incidence, duration, and severity of neutropenia, its prophylactic use for acute myeloid leukemia (AML) remains controversial due to a theoretically increased risk of relapse. The present study investigated the effects of G-CSF as primary prophylaxis for AML with remission induction therapy. A detailed literature search for related studies was performed using PubMed, Ichushi-Web, and the Cochrane Library. Data were independently extracted and assessed by two reviewers. A qualitative analysis of pooled data was conducted, and the risk ratio with corresponding confidence intervals was calculated in the meta-analysis and summarized. Sixteen studies were included in the qualitative analysis, nine of which were examined in the meta-analysis. Although G-CSF significantly shortened the duration of neutropenia, primary prophylaxis with G-CSF did not correlate with infection-related mortality. Moreover, primary prophylaxis with G-CSF did not affect disease progression/recurrence, overall survival, or adverse events, such as musculoskeletal pain. However, evidence to support or discourage the use of G-CSF as primary prophylaxis for adult AML patients with induction therapy remains limited. Therefore, the use of G-CSF as primary prophylaxis can be considered for adult AML patients with remission induction therapy who are at a high risk of infectious complications.

## Introduction

Although it only accounts for 1% of cancers, acute myeloid leukemia (AML) is the most common type of acute leukemia in adults [1]. AML is a type of blood cancer that affects blood cells in the body. When cancer occurs in blood, it generally induces the excessive reproduction of leukemic cells and a reduction in normal white blood cells. The aim of initial induction therapy for AML is a rapid reduction in leukemic cells to promote bone marrow recovery and the production of healthy blood cells. However, this antileukemic therapy damages healthy as well as leukemic cells. Neutropenia and febrile neutropenia (FN) are the most common complications of induction therapy for AML [2]. Induction therapy is also associated with a risk of life-threatening infections as well as chemotherapy delays and dose reductions that may compromise treatment outcomes.

Granulocyte colony-stimulating factor (G-CSF) reduces the incidence, duration, and severity of neutropenia [3]. However, its prophylactic use for AML remains controversial due to a theoretically increased risk of relapse because AML cells express G-CSF receptors (G-CSFRs) on their surface. Exposure to G-CSF or other myeloid growth factors was shown to induce the proliferation of AML cells in vitro [4]. Furthermore, altered myeloid growth factor signaling pathways have been suggested to play a role in leukemogenesis by providing leukemic cells with a proliferative advantage or blocking granulocytic differentiation [5].

These clinical questions need to be answered in an evidence-based manner. Therefore, we herein performed a systematic literature review to examine the effects of primary prophylaxis with G-CSF for AML, which will provide more precise estimates of its clinical efficacy and toxicity as well as serve as the basis for updates to clinical practice guidelines.

## Methods

### Search strategy

A systematic review of the literature was performed according to both the “Medical information network distribution service (Minds) Handbook for Clinical Practice Guideline Development 2014” [6] and “Minds Clinical Practice Guideline Development Guide 2017” [7] using PubMed, Ichushi-Web (Japanese medical bibliographic database), and the Cochrane Library databases. The search terms used in the combination of Mesh and keywords were as follows: “leukemia, myeloid, acute/drug therapy”, “granulocyte colony-stimulating factor”, “prevent*, prevention, and control”, “prophyla*”, and “first, initial, induction” in all fields. Initial screening was independently performed by two reviewers (*T.M.* and *Y.N.*) of the systematic review team based on the titles and abstracts of all articles for ineligible reports, followed by full-text screening (i.e., second screening) according to inclusion and exclusion criteria. The reasons for exclusion were recorded and duplicates were removed. Disagreements were resolved via consensus with the co-authors. These articles were examined for quality reporting data related to selection criteria, which are outlined in the section below.

### Selection criteria

Inclusion criteria were as follows: (1) studies with the design of a randomized controlled trial (RCT), non-RCT, and a cohort or case–control trial; (2) studies with an adult population diagnosed with AML; (3) studies that include patients in the treatment group who received standard intensive induction therapy (e.g., with the “7 + 3” regimen or a regimen of similar or higher intensity). Exclusion criteria included guidelines, reviews, letters, abstracts without an article, laboratory studies, systematic reviews, meta-analyses, and gray literature.

### Data extraction and quality assessment

After second screening, the reviewer (*T.M.*) of the systematic review team reassessed the articles and then extracted data using standardized data abstraction forms. The evidence indicated by individual studies related to critical outcomes included within the clinical questions made by the guideline creation team was divided into groups based on study design and quality. These outcome indicators included the duration of neutropenia or thrombocytopenia, infection-related mortality, disease progression/recurrence, overall survival (OS), or adverse events, such as musculoskeletal pain. Outcomes by the population, intervention, comparator, and outcome (PICO) framework on both the benefits and harm of prophylactic G-CSF were decided by the authors. Conflicts and questions were resolved by the leader (*S.Y.*) of the guideline creation team. The level of evidence was evaluated not for individual references, but by each outcome for studies grouped by study design. The certainty of evidence was assessed by the risk of bias, inconsistency, imprecision, indirectness, and publication bias. The literature quality and body of evidence were evaluated using the Grading of Recommendations, Assessment, Development, and Evaluation (GRADE) approach and then classified into four levels: “strong”, “medium”, “weak”, and “very weak”.

### Statistical methodology

The software Review Manager (RevMan, The Cochrane Collaboration, London, UK) version 5.41 was used for statistical analyses. After a qualitative analysis using *Excel*, studies were eligible for inclusion in the meta-analysis if the study design was an RCT that compared the use of G-CSF for primary prophylaxis against a non-administration control group. The risk ratio (RR) for each of the desired endpoints was calculated, and the effect size was expressed as the 95% confidence interval (CI) for each study. They were calculated using fixed- or random-effect models depending on the level of heterogeneity. A Forest plot was used to graphically represent the results of the calculated RR for individual studies and overall meta-analyses. The degree of heterogeneity was assessed using the *I*^2^ test and chi-square-based Q test. A *p*-value < 0.05 in the *Z* test was considered to be significant. A funnel plot was applied to graphically investigate the potential for a publication bias.

## Results

### Literature search

**Fig. 1:**
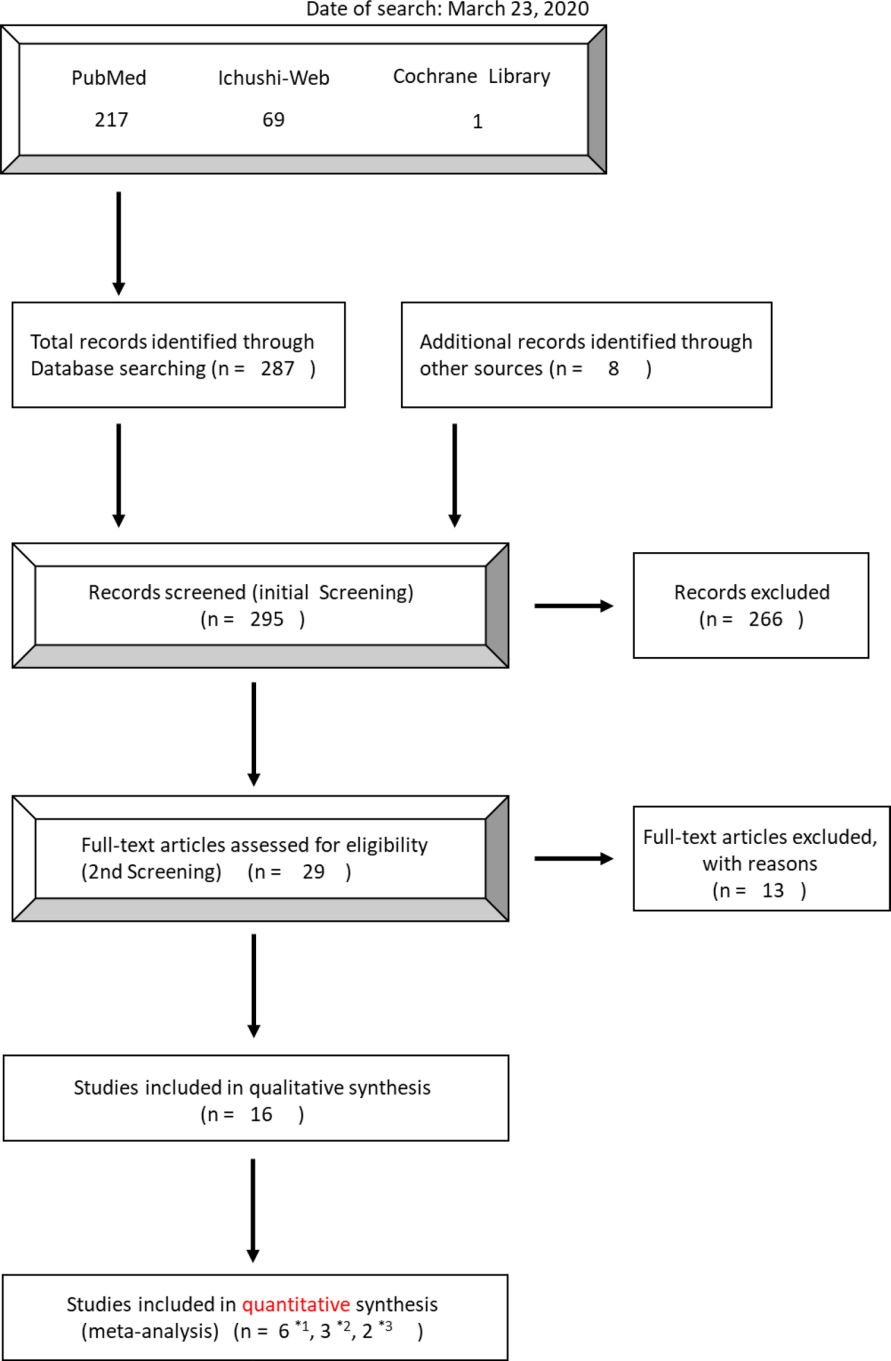
Modified PRISMA flow diagram of the literature search process. Each study was used in the meta-analysis of infection-related mortality (*1), disease progression/recurrence (*2), and adverse events, such as musculoskeletal pain (*3). PRISMA preferred reporting items for systematic reviews and meta-analysis

The initial search yielded 287 results as follows: PubMed, 217; the Cochrane Library, 1; Ichushi-Web, 69 (Date of the search: March 23, 2020). An additional 8 articles were hand-search selected and added. Among the 295 articles obtained, 279 were excluded after being screened for the following criteria: human subjects only, publication date ranging from 1st January 1990 to 31st December 2019, publications in English or Japanese, and selection criteria, which are outlined in the section above, yielding 16 articles (Fig. 1). The main reason for exclusion was related to the eligibility of subjects.

### Studies selected for the meta-analysis

Sixteen studies [3, 8–22]: 12 RCTs, 3 case–control studies, and 1 cohort study, were included in the descriptive qualitative analysis, of which 9 RCTs [3, 8–15] were examined in the meta-analysis. The 9 RCTs were published between 1990 and 2011. Meta-analyses of the study findings on the duration of neutropenia or OS were not feasible because of differences in treatment benefit and harm assessment measurements. Three RCTs [11, 12, 16] were excluded from the pooled quantitative analysis of disease progression/recurrence because of differences in assessment measurements.

Six RCTs [8–13] were ultimately selected for the meta-analysis of infection-related mortality, 3 [3, 14, 15] for disease progression/recurrence, and 2 [9, 11] for adverse events, such as musculoskeletal pain.

### Relationships between outcomes by the PICO framework and G-CSF in AML

#### Relationship between infection-related mortality and G-CSF

**Fig. 2:**
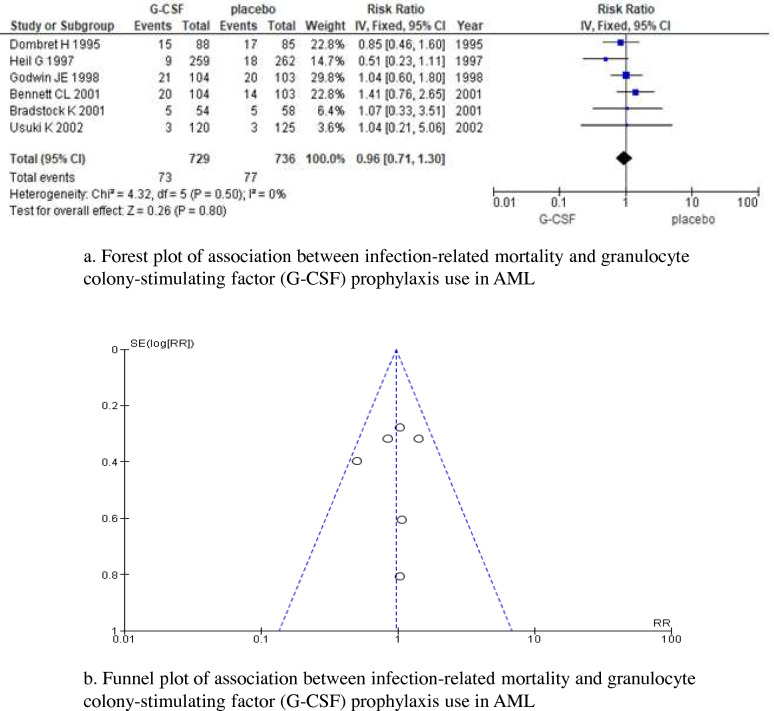
Infection-related mortality. (**a**) Forest plot and (**b**) funnel plot. A Forest plot and funnel plot of the risk ratio (RR) of infection-related mortality comparing granulocyte colony-stimulating factor (G-CSF) prophylaxis and control study arms for each study. The plot shows treatment effects versus the study size estimated from the standard error (SE) of log (RR). Open circles indicate individual studies in this meta-analysis. The broken line is a pseudo 95% confidence interval of effect measures in the study. A funnel plot showing the symmetrical distribution of studies indicating the absence of a publication bias. CI confidence interval, RR risk ratio, SE standard error

A total of 1465 patients were included in the 6 RCTs: an RCT on 112 patients aged 60 years and younger who received high-dose cytarabine [8], 2 RCTs on 766 patients aged 16 years and over-treated with standard cytarabine plus anthracycline induction therapy [9, 10], and 3 RCTs on 97 mainly elderly patients aged 55 or 65 years and older [11–13]. No significant differences were observed in infection-related mortality between patients who received primary prophylaxis with G-CSF and those who did not, with non-significant heterogeneity (RR, 0.96 [95% CI, 0.71–1.30], *p* = 0.80; *I*^2^ = 0%, *p* = 0.50) (Fig. 2a). No significant asymmetry of the funnel plot was detected (Fig. 2b). According to the GRADE approach, the quality/certainty of evidence for this outcome was “strong”.

#### Relationship between OS and G-CSF

Data from 6 RCTs [8, 11, 12, 14–16] and 1 case–control study [17] were available for inclusion in the qualitative analysis. A total of 1719 patients were included in the 6 RCTs: an RCT on 599 patients of all ages [14], 2 RCTs on 374 patients younger than 65 years [8, 15], and 3 RCTs on 746 elderly patients aged 55–65 years or older [11, 12, 16]. Although a meta-analysis of this outcome was not performed due to the difference in effect measures, 6 RCTs, as well as 1 case–control study, reached the same conclusion of no significant difference in OS due to primary prophylaxis using G-CSF. The quality/certainty of evidence was “medium”.

#### Relationship between the duration of neutropenia or thrombocytopenia and G-CSF

Data from 3 RCTs [8, 14, 16], 1 case–control study [17], and 1 cohort study [18] were available for inclusion in the qualitative analysis. Due to the difference in effect measures, we did not conduct a meta-analysis of this outcome. A total of 887 patients were included in the 3 RCTs: an RCT on 599 patients of all ages [14], an RCT on 112 patients aged 65 years and younger [8], and an RCT on 176 patients aged 61 years and older [16]. All 3 RCTs were limited due to insufficient allocation concealment and 2 out of the 3 RCTs lacked adequate blinding. In the case–control study, the ratio of the adverse cytogenetic risk group was higher in the control group than in the intervention group. There were also significant differences in age and the score of the Charlson comorbidity index between the two arms. In the cohort study, there was an unsatisfactory adjustment for multivariate prognostic variables and it may have been confounded by unmeasured variables in the relationship between the cytogenetic risk status and outcomes among patients. Nevertheless, all studies showed a significant difference in the duration of neutropenia due to the use of G-CSF as primary prophylaxis. Although none of the five studies examined the appearance of leukemic cells or changes to the number of remaining blasts in peripheral blood depending on the G-CSF stimulation, G-CSF prophylaxis against neutropenia may be beneficial for specific patients (e.g., with severe infections, the unfit, or elderly). On the other hand, prophylactic G-CSF did not significantly affect the duration of thrombocytopenia [8, 14, 16]. The quality/certainty of evidence on this outcome was “medium”.

#### Relationship between disease progression/recurrence and G-CSF

**Fig. 3:**
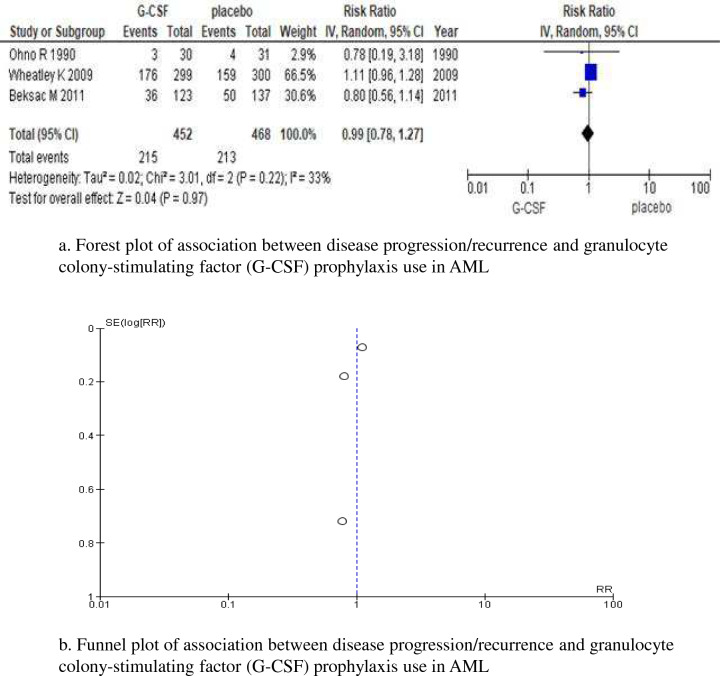
Disease progression/recurrence. (**a**) Forest plot and (**b**) funnel plot. A Forest plot and funnel plot of the risk ratio (RR) of disease progression/recurrence comparing granulocyte colony-stimulating factor (G-CSF) prophylaxis and control study arms for each study. There were too few studies and insufficient variations in standard errors to assess whether funnel plots were symmetric. However, there was no asymmetry visible in any of the funnel plots. CI confidence interval, RR risk ratio, SE standard error

Data from the following 8 studies were included in the qualitative analysis (*n* = 1752): 2 RCTs on 660 patients of all ages [3, 14], an RCT on 260 patients younger than 65 years [15], 3 RCTs on 746 elderly patients aged 55–65 years or older [11, 12, 16], a case–control study on 186 patients [17], and a cohort study on 25 patients [18]. All studies reported no significant difference in disease progression/recurrence due to the use of G-CSF as primary prophylaxis. Three out of the 6 RCTs were excluded due to differences in assessment measurements, and the remaining 3 (*n* = 920) [3, 14, 15] were included in the meta-analysis. There was no significant difference in disease progression/recurrence between patients who received primary prophylaxis with G-CSF and those who did not, with low heterogeneity (RR, 0.99 [95% CI, 0.78–1.27], *p* = 0.97; *I*^2^ = 33%, *p* = 0.22) (Fig. 3a). Although the possibility of a publication bias was not denied by the small number of studies, no apparent asymmetry of the funnel plot was detected (Fig. 3b). The quality/certainty of evidence on this outcome was “strong”.

#### Relationship between adverse events, such as musculoskeletal pain, and G-CSF

**Fig. 4:**
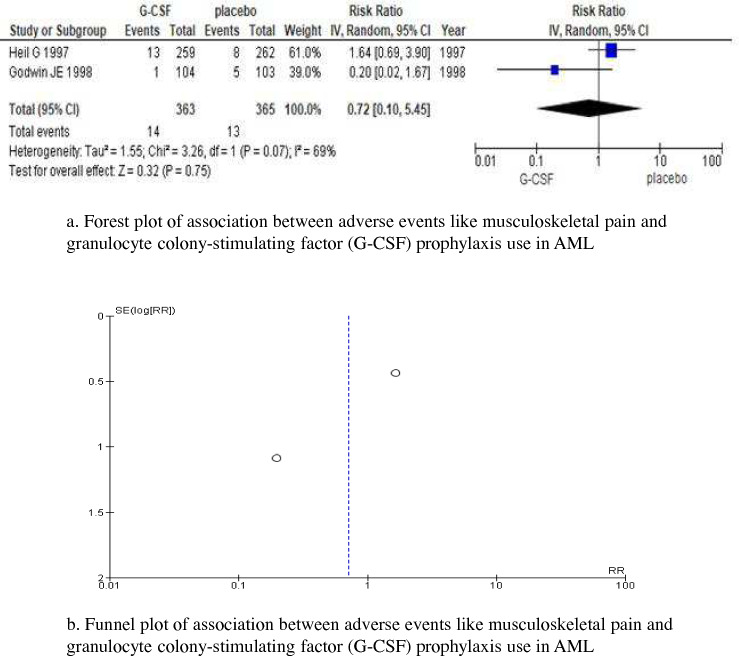
Adverse events, such as musculoskeletal pain. (**a**) Forest plot and (**b**) funnel plot. A Forest plot and funnel plot of the risk ratio (RR) of adverse events, such as musculoskeletal pain, comparing granulocyte colony-stimulating factor (G-CSF) prophylaxis and control study arms for each study. Although there were too few studies and insufficient variations in standard errors (SE) to assess whether funnel plots were symmetric, there was no asymmetry visible in the funnel plots. CI confidence interval, RR risk ratio, SE standard error

There were only two studies on this outcome. The following two RCTs were included in the meta-analysis (*n* = 728): an RCT on 521 patients of all ages [9] and 207 patients aged 55 years or older [11]. There was no significant difference in adverse events, such as musculoskeletal pain, between patients who received primary prophylaxis with G-CSF and those who did not, with heterogeneity (RR, 0.72 [95% CI, 0.10–5.45], *p* = 0.75; *I*^2^ = 69%, *p* = 0.07) (Fig. 4a). Although there was a limitation due to the small number of studies, the funnel plot indicated no publication bias (Fig. 4b). The quality/certainty of evidence was “middle”.

## Discussion

AML is a heterogeneous hematologic cancer that is characterized by different cytogenetics with different risk profiles. It evolves by the malignant transformation and clonal expansion of hematopoietic stem cells or their progenitor cells. The newly released proposal from the 5th edition of the WHO Classification and the 2022 International Consensus Classification emphasizes a genetic basis for defining diseases in AML [23, 24]. In clinical practice, a cytogenetic analysis has become essential for not only a disease diagnosis, but also its classification, prognostic stratification, and treatment strategy. Chromosomal abnormalities and the gene mutation status are the most important prognostic factors in AML for predicting the remission rate, relapse, and OS. While advances are being achieved in therapeutic approaches that target molecular abnormalities, intensive induction therapy with cytarabine and anthracycline, developed in the early 1970s, remains the standard of care for fit patients with AML. The well-known standard combination is the “7 + 3” regimen, with a 7-day continuous infusion of cytarabine on days 1–7 and anthracycline on days 1–3 [25]. The main purpose of induction therapy is to safely bring patients into complete remission without severe treatment-related toxicities or mortality. Despite advances in supportive care, the major causes of mortality in patients with AML are infectious complications because intensive induction chemotherapy lowers the white blood cell count and disrupts the immune system. Furthermore, risk assessments of the severity of infection require a detailed understanding of a patient’s host factors as well as the intensity of chemotherapy. The National Comprehensive Cancer Network and the American Society of Clinical Oncology guidelines on the use of white blood cell growth factors both state that in patients receiving chemotherapy regimens with a 10–20% risk of FN, additional risk factors (e.g., age ≥ 65 years, a decreased performance status, a history of FN, and comorbidities, including renal or liver dysfunction) need to be considered for G-CSF therapy [26, 27], most of which have been confirmed as independent risk factors for neutropenic complications in the risk model developed by Lyman et al. [28].

In the present systematic review, we found a significant difference in the duration of chemotherapy-induced neutropenia between patients who received primary prophylaxis with G-CSF and those who did not. The results of the meta-analysis also revealed that the use of G-CSF for primary prophylaxis did not correlate with infection-related mortality; however, it significantly shortened the duration of neutropenia. Moreover, the use of G-CSF as prophylaxis did not affect disease progression/recurrence, OS, or adverse events, such as musculoskeletal pain. However, the adverse events associated with the use of G-CSF as prophylaxis were too few to be fully assessed.

The functional activity of G-CSF is mediated through G-CSFRs, which play crucial roles in the proliferation and differentiation of myeloid progenitors, leading to the development of neutrophils. Since leukemic cells in AML may be stimulated via G-CSFRs, physicians are skeptical about the use of G-CSF as prophylaxis based on in vitro data. The number of cell-surface G-CSFRs differs from patient to patient, and a correlation was not observed between the expression of these receptors and leukemia morphological subtypes or cell surface markers [29]. G-CSFRs, also known as cluster of differentiation 114 (CD114), are encoded by the human *CSF3R* gene. In response to G-CSF, G-CSFRs form homodimers and activate several signal transduction pathways, including JAK/STAT, Ras/Raf/MAPK, and PKB/Akt [30]. The human *CSF3R* gene is made up of 17 exons that give rise to 7 different mRNA isoforms, labeled Class I through VII [31]. Among them, Class IV G-CSFRs are prominently expressed in patients with AML and have been linked to an increased incidence of relapse in children and adolescents with AML [32].

With advances in cancer epigenetics, hypomethylating agents were found to be beneficial for elderly AML patients [33]. Furthermore, a recent trial, phase 3 VIALE-A revealed that venetoclax plus azacitidine prolonged OS significantly more than a placebo plus azacitidine in untreated AML patients who were unsuitable candidates for standard induction therapy [34]. This combination of BCL-2 inhibitors and hypomethylating agents is the standard of care for elderly and unfit AML patients. According to a post hoc analysis from the VIALE-A trial, G-CSF was frequently used per institutional practices post-remission to manage neutropenia. The use of G-CSF was associated with shorter durations of Grade 3–4 neutropenia and FN with its use first post-remission, without evidence of a negative impact on OS [35]. However, further studies are needed due to the lack of evidence on the benefits of using G-CSF as primary prophylaxis in this setting.

Overall, the results of this systematic review and meta-analysis confirm and update previous findings on the efficacy and safety of G-CSF. However, several limitations need to be considered. Heterogeneity existed in the time to recovery of the absolute neutrophil count (ANC) or the depth of the ANC nadir. Furthermore, due to older literature in reviewed articles, different baseline characteristics, such as chromosomal abnormalities or the gene mutation status, may have affected the outcomes of patients’ responses to induction therapy and produced heterogeneity in clinical outcomes. Moreover, most articles lacked a further distinction between groups by the number expressing G-CSFRs or differences in the isoforms of G-CSFRs, thereby limiting stratified analyses. In addition, most of the literature retrieved in the present study was more than 10 years old. Recent prospective randomized trials in this area have not investigated the clinical significance of using G-CSF because the risk of FN is associated with the dose intensity of the treatment regimen and is higher in AML than in other tumors. Furthermore, the benefits of G-CSF include not only improvements in the prognosis of patients with risk factors by preventing FN, but also non-clinical aspects, such as its social contribution by allowing patients to return to society as well as the social benefit of reducing the burden on patients’ caregivers. A recent economic analysis suggested the potential of G-CSF as a cost-saving treatment when the risk of FN is approximately 17–20% [36]. Therefore, recent studies on AML in this area are limited.

## Conclusions

Primary prophylaxis with G-CSF did not correlate with infection-related mortality in adult AML patients receiving remission induction therapy; however, G-CSF significantly shortened the duration of neutropenia. Furthermore, primary prophylaxis with G-CSF did not affect disease progression/recurrence, OS, or adverse events, such as musculoskeletal pain. Therefore, the use of G-CSF as a primary prophylactic during induction therapy only needs to be considered for adult AML patients who are at a high risk of infectious complications.

## Data Availability

Data associated with this systematic review may be accessed from the corresponding author upon reasonable request.

## References

[1] Siegel RL, Miller KD, Wagle NS (2023). Cancer statistics, 2023. CA Cancer J Clin.

[2] Peseski AM, McClean M, Green SD (2021). Management of fever and neutropenia in the adult patient with acute myeloid leukemia. Expert Rev Anti Infect Ther.

[3] Ohno R, Tomonaga M, Kobayashi T (1990). Effect of granulocyte colony-stimulating factor after intensive induction therapy in relapsed or refractory acute leukemia. N Engl J Med.

[4] Avalos BR, Lazaryan A, Copelan EA (2011). Can G-CSF cause leukemia in hematopoietic stem cell donors?. Biol Blood Marrow Transplant.

[5] De Figueiredo LL, De Abreu e Lima RS, Rego EM (2004). Granulocyte colony-stimulating factor and leukemogenesis. Mediators Inflamm.

[6] Morizane T, Yoshida M, Kojimahara N et al (2014) Minds handbook for clinical practice guideline development 2014. Japan Council for Quality Health Care, Tokyo. https://minds.jcqhc.or.jp/s/developer_manual (in Japanese)

[7] Kojimahara N, Nakayama T, Morizane T et al (2017) Minds manual for guideline development 2017. Japan Council for Quality Health Care, Tokyo. https://minds.jcqhc.or.jp/s/developer_manual (in Japanese)

[8] Bradstock K, Matthews J, Young G (2001). Effects of glycosylated recombinant human granulocyte colony-stimulating factor after high-dose cytarabine-based induction chemotherapy for adult acute myeloid leukaemia. Leukemia.

[9] Heil G, Hoelzer D, Sanz MA (1997). A randomized, double-blind, placebo-controlled, phase III study of filgrastim in remission induction and consolidation therapy for adults with de novo acute myeloid leukemia. The International Acute Myeloid Leukemia Study Group. Blood.

[10] Usuki K, Urabe A, Masaoka T (2002). Efficacy of granulocyte colony-stimulating factor in the treatment of acute myelogenous leukaemia: a multicentre randomized study. Br J Haematol.

[11] Godwin JE, Kopecky KJ, Head DR (1998). A double-blind placebo-controlled trial of granulocyte colony-stimulating factor in elderly patients with previously untreated acute myeloid leukemia: a Southwest oncology group study (9031). Blood..

[12] Dombret H, Chastang C, Fenaux P (1995). A controlled study of recombinant human granulocyte colony-stimulating factor in elderly patients after treatment for acute myelogenous leukemia. AML Cooperative Study Group. N Engl J Med.

[13] Bennett CL, Hynes D, Godwin J (2001). Economic analysis of granulocyte colony stimulating factor as adjunct therapy for older patients with acute myelogenous leukemia (AML): estimates from a Southwest Oncology Group clinical trial. Cancer Invest.

[14] Wheatley K, Goldstone AH, Littlewood T (2009). Randomized placebo-controlled trial of granulocyte colony stimulating factor (G-CSF) as supportive care after induction chemotherapy in adult patients with acute myeloid leukaemia: a study of the United Kingdom Medical Research Council Adult Leukaemia Working Party. Br J Haematol.

[15] Beksac M, Ali R, Ozcelik T (2011). Short and long term effects of granulocyte colony-stimulating factor during induction therapy in acute myeloid leukemia patients younger than 65: results of a randomized multicenter phase III trial. Leuk Res.

[16] Amadori S, Suciu S, Jehn U (2005). Use of glycosylated recombinant human G-CSF (lenograstim) during and/or after induction chemotherapy in patients 61 years of age and older with acute myeloid leukemia: final results of AML-13, a randomized phase-3 study. Blood.

[17] Kang KW, Kim DS, Lee SR (2017). Effect of granulocyte colony-stimulating factor on outcomes in patients with non-M3 acute myelogenous leukemia treated with anthracycline-based induction (7+3 regimen) chemotherapies. Leuk Res.

[18] Kawato M, Mikuni C, Hirota Y (2003). Effect of Granulocyte colony-stimulating factor on neutropenia after chemotherapy in acute myeloid leukemia. Iryo.

[19] Standaert B, Goldstone J, Lu ZJ (2002). Economic analysis of filgrastim use for patients with acute myeloid leukaemia in the UK: a comparison of collection methods of resource use data. Pharmacoeconomics.

[20] Harousseau JL, Wu D (1995). The use of GM-CSF and G-CSF in the treatment of acute leukemias. Leuk Lymphoma.

[21] Bradley AM, Deal AM, Buie LW (2012). Neutropenia-associated outcomes in adults with acute myeloid leukemia receiving cytarabine consolidation chemotherapy with or without granulocyte colony-stimulating factor. Pharmacotherapy.

[22] Maslak PG, Weiss MA, Berman E (1996). Granulocyte colony-stimulating factor following chemotherapy in elderly patients with newly diagnosed acute myelogenous leukemia. Leukemia.

[23] Khoury JD, Solary E, Abla O (2022). The 5th edition of the World Health Organization classification of haematolymphoid tumours: myeloid and histiocytic/dendritic neoplasms. Leukemia.

[24] Arber DA, Orazi A, Hasserjian RP (2022). International consensus classification of myeloid neoplasms and acute leukemias: integrating morphologic, clinical, and genomic data. Blood.

[25] Bernard J, Weil M, Boiron M (1973). Acute promyelocytic leukemia: results of treatment by daunorubicin. Blood.

[26] National Comprehensive Cancer Network (2023) Hematopoietic growth factors. https://www.nccn.org/professionals/physician_gls/pdf/growthfactors.pdf. (Version 2; 2023). Accessed Oct 2023

[27] Smith TJ, Bohlke K, Lyman GH (2015). Recommendations for the use of WBC growth factors: American Society of Clinical Oncology clinical practice guideline update. J Clin Oncol.

[28] Lyman GH, Kuderer NM, Crawford J (2011). Predicting individual risk of neutropenic complications in patients receiving cancer chemotherapy. Cancer.

[29] Shinjo K, Takeshita A, Ohnishi K (1997). Granulocyte colony-stimulating factor receptor at various differentiation stages of normal and leukemic hematopoietic cells. Leuk Lymphoma.

[30] Theyab A, Algahtani M, Alsharif KF (2021). New insight into the mechanism of granulocyte colony-stimulating factor (G-CSF) that induces the mobilization of neutrophils. Hematology.

[31] Mehta HM, Futami M, Glaubach T (2014). Alternatively spliced, truncated GCSF receptor promotes leukemogenic properties and sensitivity to JAK inhibition. Leukemia.

[32] Ehlers S, Herbst C, Zimmermann M (2010). Granulocyte colony-stimulating factor (G-CSF) treatment of childhood acute myeloid leukemias that overexpress the differentiation-defective G-CSF receptor isoform IV is associated with a higher incidence of relapse. J Clin Oncol.

[33] Kantarjian HM, Thomas XG, Dmoszynska A (2012). Multicenter, randomized, open-label, phase III trial of decitabine versus patient choice, with physician advice, of either supportive care or low-dose cytarabine for the treatment of older patients with newly diagnosed acute myeloid leukemia. J Clin Oncol.

[34] DiNardo CD, Jonas BA, Pullarkat V (2020). Azacitidine and venetoclax in previously untreated acute myeloid leukemia. N Engl J Med.

[35] Pratz KW, DiNardo CD, Selleslag D (2022). Postremission cytopenia management in patients with acute myeloid leukemia treated with venetoclax and azacitidine in VIALE-A. Am J Hematol.

[36] Ramsey SD, Liu Z, Boer R (2009). Cost-effectiveness of primary versus secondary prophylaxis with pegfilgrastim in women with early-stage breast cancer receiving chemotherapy. Value Health.

